# Distribution, Assessment, and Source of Heavy Metals in Sediments of the Qinjiang River, China

**DOI:** 10.3390/ijerph19159140

**Published:** 2022-07-26

**Authors:** Shuncun Zhang, Bo Chen, Junru Du, Tao Wang, Haixin Shi, Feng Wang

**Affiliations:** 1 Northwest Institute of Eco-Environment and Resources, Chinese Academy of Sciences, Lanzhou 730000, China; zhangshuncun@126.com (S.Z.); wangtao20200202@163.com (T.W.); 2 Guangxi Key Laboratory of Green Chemical Materials and Safety Technology, Beibu Gulf University, Qinzhou 515000, China; djr572899639@163.com (J.D.); shihaixin2006@163.com (H.S.); 3 University of Chinese Academy of Sciences, Beijing 100049, China

**Keywords:** heavy metals, risk assessment, source analysis, surface sediment, Qinjiang River

## Abstract

Heavy metals are toxic, persistent, and non-degradable. After sedimentation and adsorption, they accumulate in water sediments. The aim of this study was to assess the extent of heavy metal pollution of Qinjiang River sediments and its effects on the ecological environment and apportioning sources. The mean total concentrations of Mn, Zn, Cr, Cu, and Pb are 3.14, 2.33, 1.39, 5.79, and 1.33 times higher than the background values, respectively. Co, Ni, and Cd concentrations are lower than the background values. Fe, Co, Ni, Cd, Cr, Cu, and Pb are all primarily in the residual state, while Mn and Zn are primarily in the acid-soluble and oxidizable states, respectively. Igeo, *RI*, SQGs, and *RAC* together indicate that the pollution status and ecological risk of heavy metals in Qinjiang River sediments are generally moderate; among them, Fe, Co, Ni, Cd, Cr, and Pb are not harmful to the ecological environment of the Qinjiang River. Cu is not readily released because of its higher residual composition, suggesting that Cu is less harmful to the ecological environment. Mn and Zn, as the primary pollution factors of the Qinjiang River, are harmful to the ecological environment. This heavy metal pollution in surface sediments of the Qinjiang River primarily comes from manganese and zinc ore mining. Manganese carbonate and its weathered secondary manganese oxide are frequently associated with a significant amount of residual copper and Cd, as a higher pH is suitable for the deposition and enrichment of these heavy metals. Lead–zinc ore and its weathering products form organic compounds with residual Fe, Co, Cr, and Ni, and their content is related to salinity. The risk assessment results of heavy metals in sediments provide an important theoretical basis for the prevention and control of heavy metal pollution in Qinjiang River.

## 1. Introduction

Sediments are a crucial part of rivers and lakes. During several physical and chemical processes, suspended solids and different ions in the water are adsorbed and enriched in sediments in river channels, reducing the contamination in water sediments [1,2,3]. This can objectively show the area’s water quality. Unlike other pollutants in water bodies, heavy metal contaminants cannot be efficiently eliminated by natural decomposition processes and instead accumulate in sediments in different ways [4,5,6]. In most cases, more than 95% of heavy metals in water bodies are eliminated and stored in sediments in various forms [7,8,9]. Thus, there is a continuous accumulation process of heavy metals in sediments. The sediments in the water body are the “sinks” of heavy metal pollutants in the water body [10,11]. When the environmental medium conditions (such as pH, Eh) change, the heavy metals in the sediments can be released into the water body and become the water body’s “secondary pollution source” [12,13,14]. Heavy metals in the bottom sediments of water bodies have extensive sources, simple accumulation, and long residual time and are difficult to detect after pollution [15,16]. Generally, heavy metals in sediments are present in various fractions (acid-soluble, reducible, oxidizable, and residual) [17,18], and the fraction content influences the heavy metals’ bioavailability; for example, the residual is more stable and less likely to be released. Thus, the total concentrations of heavy metals in sediments do not exactly show the environmental pollution status. Additionally, it must be determined in combination with the geochemical fractions of heavy metals in sediments [19,20].

Qinzhou City is located in the southernmost part of the Qinhang metallogenic belt [21]. There are several ilmenite, manganese, and lead–zinc ore fields in Qinzhou. Medium-sized and large metal ores include Xinhua lead–zinc ore in Pubei, Huarong-Dadong manganese ore, and Nahualing manganese ore in Qinnan District [22]. With the implementation of regional economic development policies, including the Belt and Road Initiative, free trade zones, and world-class petrochemical industrial parks, Qinzhou’s economy has rapidly developed, and the degree of industrialization and urbanization has been continuously promoted, bringing extensive pressure on environmental quality [23,24,25]. Recently, the water quality of monitored sections of the Qinjiang River has typically been classified as inferior V, indicating serious pollution. However, few studies have focused on the ecological risks of sediment pollution in the Qinjiang River; therefore, it is urgent and crucial to perform research work related to heavy metal pollution in Qinjiang River sediments.

This study primarily addresses the environmental pollution levels and feasible sources of heavy metals (Fe, Mn, Zn, Co, Ni, Cd, Cr, Cu, and Pb) in the sediments of the Qinjiang River. The heavy metals were categorized into four different fractions using the improved BCR sequential extraction method, and then the ecological risk was evaluated using geo-accumulation index (I_geo_), potential ecological risk index (*RI*), sediment quality guidelines (SQGs), and risk assessment code (*RAC*), and finally, the sources of heavy metals were examined using principal component analysis and geological tracing of metallic ore. The combination of statistical analysis and each index can offer a comprehensive understanding of the heavy metal risks of Qinjiang River sediments and can be employed to provide a scientific basis for environmental management and environmental legislation, including pollution control of Qinjiang River water bodies, substrate dredging, etc., so that relevant managers can make targeted adjustments to the regional industrial structure and formulate environmental protection methods that are more suitable to the Qinzhou City’s development stage and the Qinjiang River’s functional needs.

## 2. Materials and Methods

### 2.1. Study Area

The Qinjiang River is located in Qinzhou City, Guangxi Zhuang Autonomous Region, and belongs to the Pearl River system. It originates from Bainiuling at the eastern foot of Dongshan Mountain, Pingshan Town, Lingshan County, flows through more than half of Qinzhou City, and finally flows into the Maowei Sea from Shajing. With a total length of 195.26 km and a catchment area of 2391.34 km^2^, it is the largest river flowing into the Maowei Sea area of Qinzhou Bay. It is a crucial water source for industrial and agricultural production and life in Qinzhou City. The study area is situated in the subtropical monsoon climate zone, with abundant rainfall and usual floods and droughts.

### 2.2. Sample Collection and Pre-Treatment

**Figure 1:**
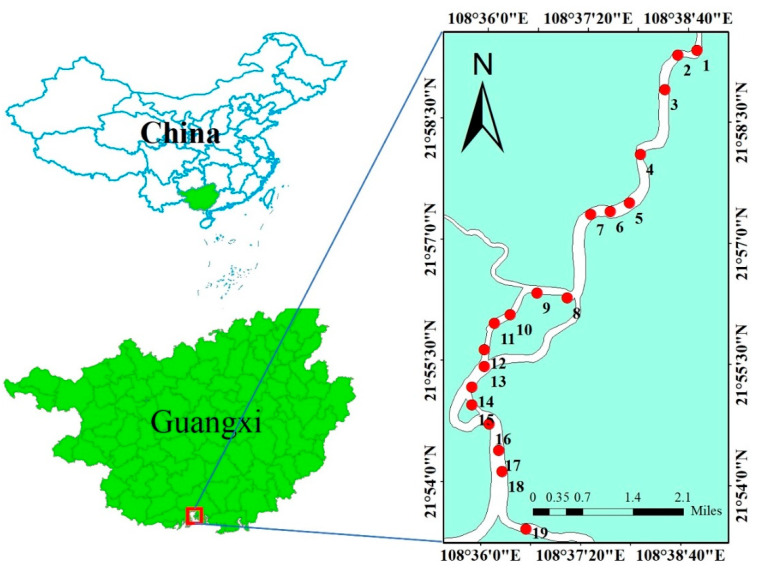
Location of the study area and sample sites.

A total of 19 stations were chosen from the upstream to the Qinjiang estuary along the Qinjiang River Basin (marked as S1 to S19) (Figure 1). The surface sediments (approximately 0–10 cm in depth) were obtained in December 2021 using a gravity sampler. All the surface sediments in contact with the sampler were cleaned with a plastic scraper to reduce the disturbance of sediment samples. Three parallel subsamples were obtained from each station. All samples were subsequently transported back to the laboratory in labeled polyethylene sealable bags and freeze-dried using a freeze drier (CHRIST Alpha2-4LSC basic). The frozen samples were then sequentially dried in natural air, dried at high temperature, ground, and passed through a 200-mesh nylon sieve to obtain the sample to be examined [26].

### 2.3. Physicochemical Analysis of Sediments

Fresh samples were mixed at a water-to-soil ratio of 1:2.5 [27] and centrifuged at 3500 rpm for 10 min; subsequently, the sediment pH and salinity were measured using a multiparameter analyzer (DZC-708). The total organic carbon (TOC) was determined by the loss-on-ignition [28]. The pretreated samples were roasted in a high-temperature muffle furnace (HT16/17, Nabertherm), and the content of organic matter (OM) in the samples was calculated according to the mass difference before and after, and the TOC content was finally converted using OM.

### 2.4. Microwave-Assisted Acid Digestion and Determination of Metals

Aliquots of ~0.1 g of sediments were placed in digestion tanks containing a 5:4:2 mixture of HNO_3_ + HF + HClO_4_. The samples were subsequently heated in microwave digestion apparatus (CEM/MARS6) for the following cycle. At 1600 W of power, the temperature was raised to 170 °C for 30 min and maintained for 20 min, followed by 210 °C for 40 min maintained for 30 min [29]. Subsequently, the digested samples were inserted into an acid purifier at 150 °C raising the acid to 1 mL. After cooling, the samples were diluted to 50 mL with ultrapure water and filtered through a 0.45 μm filter membrane, and finally, a 50 mL centrifuge tube was used to perform the test.

The concentrations of Fe, Mn, Zn, Co, Ni, Cd, Cr, Cu, and Pb in samples extracted from sequential extraction and microwave digestion were measured using ICP-OES (PE Optima8000, Crystal City, WA, USA), and the accuracy and precision of heavy metals analyses were verified using the standard reference samples (GSS-1). The mean recovery (%) in different chemical phases was 80–120%, and the relative error was generally <10%.

### 2.5. Sequential Extraction Procedure (BCR)

Acid-soluble, reducible, oxidizable, and residual fractions were extracted sequentially using the improved BCR sequential extraction method [30]. Table 1 shows the extraction agent and target sediment fraction employed in each step: Firstly, 1.00 g of sample was weighed into a 100 mL polypropylene centrifuge tube for the acid soluble fraction (F1, 0.11 mol/L CH_3_COOH), reducible fraction (F2, 0.5 mol/L NH_2_OH-HCl, pH 1.5), oxidizable fraction (F3, 1.0 mol/L CH_3_COONH_4_, pH 2.0), and residual fraction (F4, HF–HNO_3_–HClO_4_) of sediments. At the end of each extraction, the centrifuge tube was placed in a centrifuge at 4000 rpm for 10 min. The supernatant was collected, and the residue was cleaned with ultrapure water twice, transferred into a 50 mL volumetric flask, and fixed with 3% dilute nitric acid. After passing through a 45 μm filter membrane, the centrifuge tube was loaded with 50 mL for testing.

### 2.6. Contamination and Risk Assessment of Heavy Metals in Sediments

#### 2.6.1. Geo-Accumulation Index (I_geo_)

The geo-accumulation index (I_geo_) was first suggested by Müller, a scientist from the Sediment Research Institute of Heidelberg University in Germany, in 1969 [31,32]. It shows the pollution level through the heavy metal content in sediments, indicates the natural variation characteristics of the distribution of heavy metals, and determines the effect of human activities on the environment, which is a crucial parameter. It is determined by the following Equation (1):

(1) $$I_{geo}=\log_{2}\left[C_{n}/k*B_{n}\right]$$

where *C_n_* represents the determined concentration of heavy metal (mg/kg); *k* represents the correction coefficient considered due to the geological differences of rocks in various areas, which is generally 1.5; and *B_n_* denotes the heavy metal background concentration n (mg/kg). The background concentrations of heavy metals in the soil employed in this study are as follows: Mn = 159.32, Zn = 48.25, Co = 14.60, Ni = 24.00, Cd = 0.07, Cr = 21.41, Cu = 11.31, and Pb = 20.43 [33,34] (Fe rarely contaminates the environment, and therefore, there is no background value). According to the various I_geo_ values, the heavy metal pollution levels can be interpreted as follows: I_geo_, no pollution; 0 < I_geo_ ≤ 1, low pollution; 1 < I_geo_ ≤ 2, near moderate pollution; 2 < I_geo_ ≤ 3, moderate pollution; 3 < I_geo_ ≤ 4, near high pollution; 4 < I_geo_ ≤ 5, high pollution; 5 < I_geo_ ≤ 6, very high pollution.

#### 2.6.2. Potential Ecological Risk Index

The potential ecological *RI* method is a set of approaches for evaluating heavy metal pollution and ecological damage developed by the Swedish scientist Hakanson based on sedimentology [35]. It covers several research fields combining biotoxicology, environmental chemistry, and ecology. The ecological risk of heavy metals on soil is comprehensively assessed, and the potential damage degree is quantitatively categorized (Table 2). The value of *RI* was computed using the following equations:

$$C_{f}^{i}=\frac{C^{i}}{C_{n}^{i}},C_{d}=\sum C_{f}^{i},E_{r}^{i}=T_{r}^{i}C_{f}^{i},RI=\sum E_{r}^{i}$$

where $C_{f}^{i}$ represents the pollution coefficient of heavy metals *i*, $C^{i}$ represents the determined concentration of heavy metals i (mg/kg), $C_{n}^{i}$ denotes the background values of heavy metals (mg/kg), $C_{d}$ represents the sum of pollution coefficients of different heavy metals, $E_{r}^{i}$ denotes the potential ecological risk factor, RI represents the potential ecological RIs, $T_{r}^{i}$ denotes the toxicity coefficient of heavy metals, and i denotes the toxicity level of heavy metals and the organisms’ sensitivity to heavy metal pollution, and its values are Zn = 1, Cr = 2, Co = Ni = Cu = Pb = 5, and Cd = 30 [35]. According to the determined level of toxicity of the heavy metals, we also considered the high heavy metal content and biological activity in the study area, defining five categories of $E_{r}^{i}$ and four categories of RI (Table 2) [36,37]. However, other evaluation criteria have been used by Barcauskaite et al. 2020, Wu et al. 2010, Zhu et al. 2012 [38,39,40].

#### 2.6.3. Sediment Quality Guidelines

The SQGs can be employed to evaluate the heavy metal pollution level in sediments. Theoretically, SQGs are derived from the accumulation of datasets of sediment chemistry and corresponding adverse biological impacts [41,42], whereas the empirical assessment of SQGs is based on the total amounts of heavy metals in sediments [43]. There are two concentration thresholds for SQGs; one is unlikely to generate toxic reactions, and the other is likely to generate toxic reactions, and the pollutant concentrations between the two thresholds have significant uncertainty. To resolve this situation, it is crucial to conduct a site-specific analysis by observing the health and behavior of benthic organisms at the site. One frequently employed approach is to use the threshold effect level (TEL) and possible effect level (*PEL*) to compare with the heavy metal concentration to assess the degree of harmful impacts of sediment-related chemical states on benthic organisms [44,45]. These two levels defined three ranges of benthic hazards: no harm (<TEL); may cause harm (>TEL and <*PEL*); and harm (>*PEL*).

Furthermore, as heavy metals always appear in complex mixtures in sediments, their ecological risk can also be further assessed by their *PEL* and the resulting mass fraction to yield the mean probable-effect-level quotient (*mPEL*-*Q*) [46]; the formula is as follows:

$$mPEL-Q=\sum_{i=1}^{n}\left(C_{r}^{i}/PEL_{i}\right)/n$$

where $C_{r}^{i}$ represents the measured concentration of the heavy metal *i*, $PEL_{i}$ represents the *PEL* for the heavy metal *i*, and *n* is the number of heavy metal species. The *mPEL*-*Q* indices can be grouped into the following four categories: *mPEL*-*Q* ≤ 0.1, low risk; 0.1 < *mPEL*-*Q* ≤ 1, considerable risk; 1 < *mPEL*-*Q* ≤ 5, high risk; *mPEL*-*Q* > 5, very high risk.

#### 2.6.4. Risk Assessment Code

The *RAC* was employed to evaluate the bioavailability and mobility of heavy metals in sediments [47], which is closely related to the concentration of heavy metals in the sequentially extracted acidic soluble. The *RAC* equation is as follows:

$$RAC=\frac{F1}{F1+F2+F3+F4}$$

The RAC indices can be grouped into the following five categories: *RAC* < 1%, no risk; 1% < *RAC* < 10%, low risk; 11% < *RAC* < 30%, considerable risk; 31% < *RAC* < 50%, high risk; *RAC* > 50%, very high risk.

## 3. Results and Discussion

### 3.1. Total Concentration and Physicochemical Properties of Heavy Metals in Sediments of the Qinjiang River

**Figure 2:**
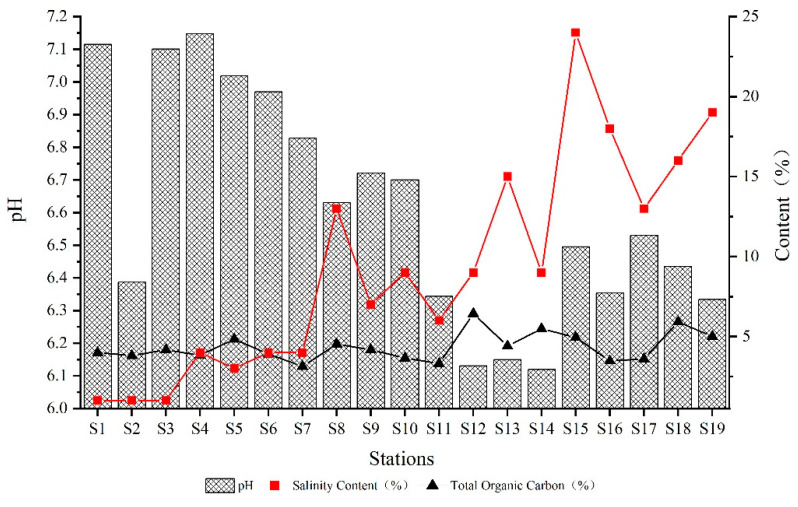
Physicochemical characteristics of sediment at the different stations.

Figure 2 presents the pH values, salinity, and TOC concentration of sediments in the Qinjiang River. The pH of sediments in the Qinjiang River ranged from 6.119 to 7.147, with a mean value of 6.61 and a weak acidity generally; the variation of salinity ranged from 0.01% to 0.24%; and the variation of ω (TOC) ranged from 3.12% to 6.43%, with a mean value of 4.43%. When the point is closer to the Qinjiang River estuary, the pH tends to decrease as a whole, and salinity and TOC content tend to increase as a whole. Remarkably, pH has considerably lower values at S2, S12, S13, and S14, probably because the sampling area is close to the industrial park and the agricultural planting area. The sediment acidity was aggravated by the acidity of the water bodies caused by the sewage discharge and waste accumulation in the sampling area’s vicinity. Furthermore, the stations with significant heavy metal concentrations also have high TOC, showing that the TOC content influences the heavy metals’ enrichment.

Table 3 summarizes the characteristics of heavy metal concentrations in sediments in the Qinjiang River. Average total heavy metal concentrations (mg/kg) were discovered in the decreasing order of Fe (31401.95) > Mn (500.27) > Zn (112.49) > Cu (65.45) > Cr (29.78) > Pb (22.99) > Ni (17.82) > Co (9.05) > Cd (0.02); Mn, Zn, Cr, Cu, and Pb had higher concentration values than their background values by 3.14, 2.33, 1.39, 5.79, and 1.13 times, respectively. High concentrations of Mn, Cd, and Cu were discovered in S5, which is situated in the city center and has hospitals, markets, and Nixing pottery factories nearby. High concentrations of Fe and Pb were discovered in S12, which has several agricultural planting areas and industrial parks. A high concentration of Cr is distributed in the dock area. High concentrations of Zn, Co, and Ni were distributed in the aquaculture area (S18). Heavy metals in waste and polluted soil are readily leached into the near-source water via runoff, and heavy metals migrate through rivers and accumulate in sediments [48]. Thus, these buildings may become the heavy metal pollution’s primary source of sediments and must be addressed in the subsequent analysis.

The coefficient of variation (CV) can show the uniformity and degree of variation of heavy metals in soil stations. Generally, the larger the CV, the greater the spatial dispersion that may be influenced by human activities. The CV of Fe, Co, Ni, Cd, Cr, Cu, and Pb ranges from 7.64% to 26.49%, less than 30%, showing that the distribution of these seven heavy metals in the watershed is relatively stable, and these heavy metals are primarily affected by natural factors. The coefficients of variation of Mn (53.55%) and Zn (37.02%) both exceeded 30%, suggesting that the spatial dispersion of Mn and Zn was greater, and it is speculated that Mn and Zn are primarily affected by human activities.

### 3.2. Geochemical Fractionations of Heavy Metals

**Figure 3:**
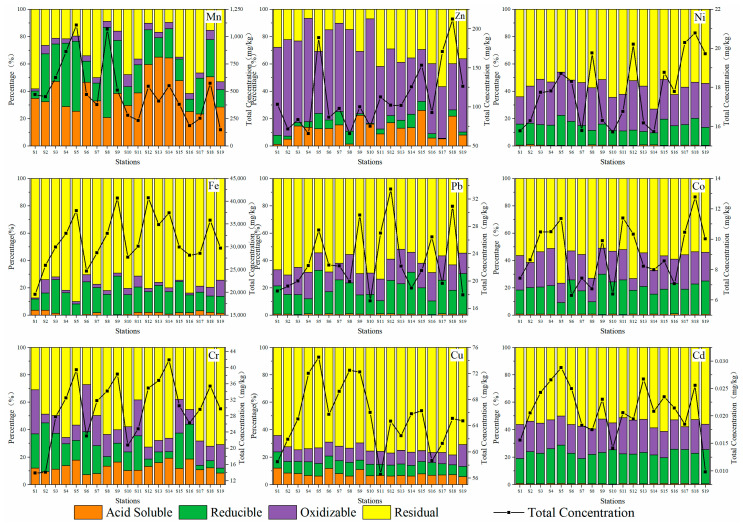
Percentage distribution of heavy metals in four geochemical fractions at 19 stations.

Figure 3 shows four geochemical fractions of nine heavy metals (Fe, Mn, Zn, Co, Ni, Cd, Cr, Cu, and Pb) in surface sediments at 19 stations in the Qinjiang River extracted using the improved BCR sequential extraction method. Therefore, different proportions of geochemical fractions of heavy metals can be grouped into the following three categories: The first category is Mn, which is primarily found in the acid-soluble fraction (F1) and accounts for 38.92%, suggesting that Mn has high bioavailability and is easily released under acidic conditions; the second category is Zn, which is mainly found in the oxidizable fraction (F3) and accounts for 54.50%. Such heavy metals primarily occur in the form of iron and manganese oxides and organically bound states, which migrate with the change of redox potential, resulting in secondary pollution of water; the third category is Fe, Co, Ni, Cd, Cr, Cu, and Pb, and these seven heavy metals are primarily found in the residual fraction (F4). The average proportion of F4 is 78.13%, 57.39%, 56.60%, 55.36%, 55.31%, 73.74%, and 62.88%, respectively, and some investigations have revealed [49] that the residual fraction of heavy metals was almost unused by organisms. This can have a specified effect on organisms only by converting them into a soluble fraction by chemical reactions. Thus, these seven heavy metals are relatively stable in the Qinjiang River sediments and cannot easily pollute the ecological environment.

### 3.3. Pollution and Risk Assessment

#### 3.3.1. Geo-Accumulation Index (I_geo_)

**Figure 4:**
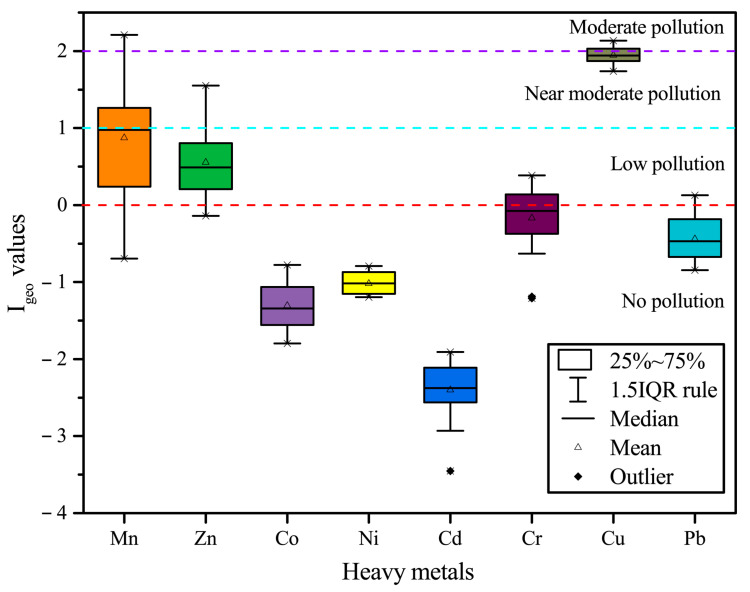
Geo-accumulation index (I_geo_) values of heavy metals in surface sediments from the Qinjiang River.

Figure 4 shows the I_geo_ values of the eight heavy metals. All heavy metals are in the decreasing order of Cu (1.94) > Mn (0.87) > Zn (0.18) > Cr (−0.17) > Pb (−0.44) > Ni (−1.02) > Co (−1.31) > Cd (−2.40). The negative I_geo_ values for Co, Ni, and Cd at all stations show a no pollution level; although the mean value of Cr and Pb is negative, there are still individual stations, which have low pollution. The mean value of Mn and Zn is between 0 and 1 (low pollution). However, at individual stations, Mn reaches 1 to 2 (moderate pollution) and 2 to 3 (moderate pollution). The mean I_geo_ value of Cu is much higher than other heavy metals, i.e., close to 2 to 3 (moderate pollution), showing that Cu is the most polluting heavy metal in the study area.

#### 3.3.2. Potential Ecological Risk Index

**Figure 5:**
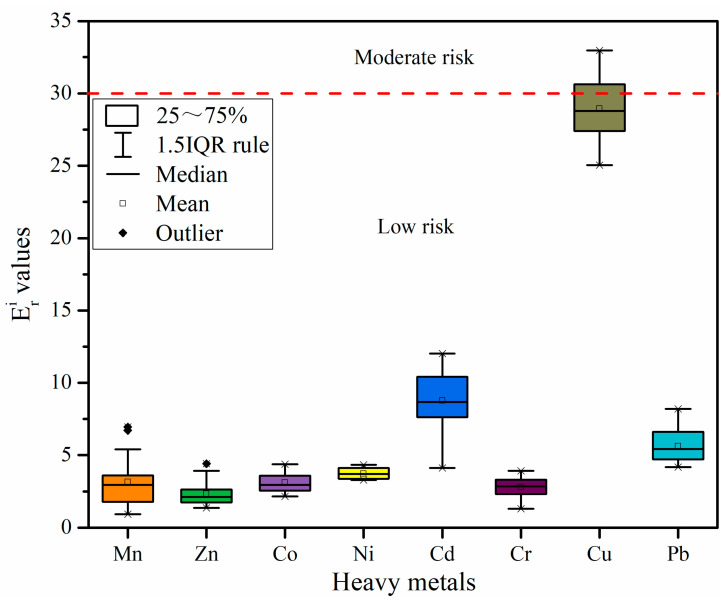
Values of potential ecological risk factor ($E_{r}^{i}$) of heavy metals in the sediments of Qinjiang River.

Figure 5 shows the values of potential ecological risk factors ($E_{r}^{i}$). The $E_{r}^{i}$ of all heavy metals were discovered in the decreasing order of Cu (28.95) > Cd (8.78) > Pb (5.63) > Ni (3.71) > Co (3.10) > Mn (3.14) > Cr (2.78) > Zn (2.33), and all heavy metals exhibited low risk. The $E_{r}^{i}$ values of Cu, Mn, Co, Ni, Cr, Cu, and Pb were consistent with the results generated by the I_geo_ values. However, Cd and Zn show a different result, which is probably because the $E_{r}^{i}$ primarily shows the heavy metal toxicity level and the organisms’ sensitivity to heavy metal pollution. When this approach is employed for risk assessment of heavy metals, the heavy metals’ toxicity coefficient has a significant influence on the results.

**Figure 6:**
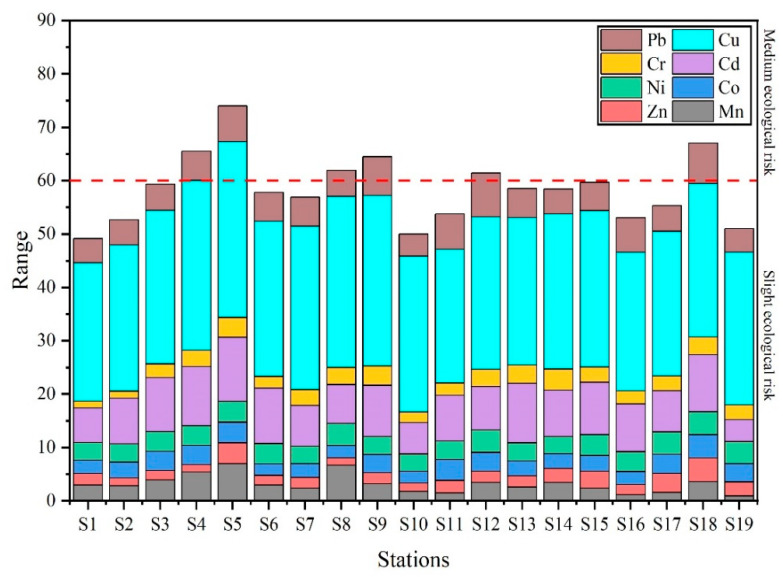
Values of potential ecological risk index (*RI*) at different stations.

Figure 6 shows the values of the potential ecological risk index (*RI*). There are six stations in the Qinjiang River with *RI* values greater than 60 (medium ecological risk), and the *RI* values of these stations were discovered in the decreasing order of S5 (67.05) > S4 (65.09) > S9 (64.47) > S8 (61.91) > S12 (61.44). Consistent with the results of the total concentration of heavy metals, higher *RI* values were discovered at S5 and S18, showing a higher ecological risk at these two stations. According to the field investigation, it is speculated that the pollution of S5 is due to intensive human activities and industrial pollution, including hospitals, markets, and Nixing pottery factories, whereas the pollution at S18 arises from the adjacent aquaculture area.

#### 3.3.3. Sediment Quality Guidelines (SQGs)

Considering the limitations of the I_geo_ and *RI* approaches, another approach was used according to SQGs, based on the heavy metals’ total concentration. In this study, a set of SQGs for the TEL and *PEL* was employed to determine the ecotoxicological implications of seven heavy metals (Zn, Ni, Cd, Cr, Cu, and Pb) in the Qinjiang River. Table 4 shows the results. The mean concentrations of Cd and Cr at all stations are less than TEL, suggesting that neither Cd nor Cr will harm benthic organisms; 19%, 21%, and 85% of Zn, Ni, and Pb samples, respectively, are lower than TEL, respectively, and 81%, 79%, and 15% are between TEL and *PEL*, showing that Zn and Ni may occasionally have harmful impacts on benthic organisms at some stations. Mn was lower than the *PEL* at most stations (94%); however, it was the only heavy metal in the study that exceeded the *PEL* at one station (S5). Thus, Mn contamination at station S5 must be addressed. The mean concentrations of Cu at all stations were between TEL and *PEL*, indicating that Cu may be harmful to benthic organisms.

**Figure 7:**
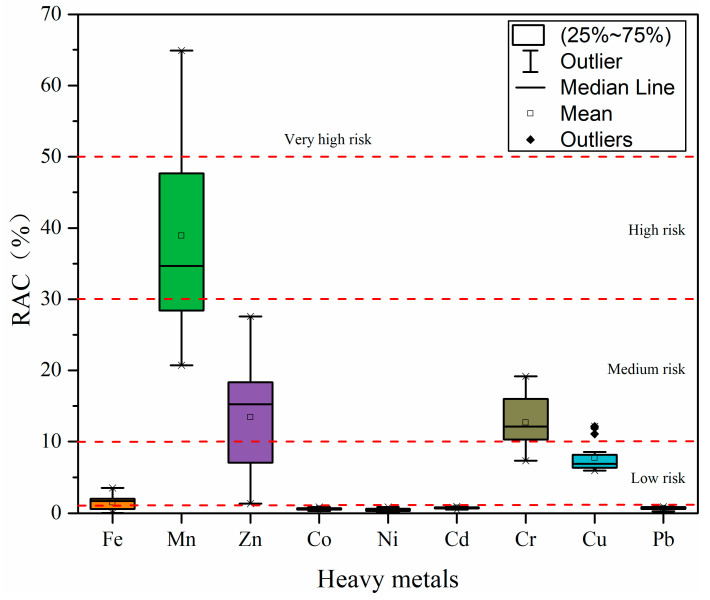
Risk assessment code (*RAC*) values of heavy metals in surface sediments from the Qinjiang River.

Additionally, the *mPEL*-*Q* values of each station were computed to further investigate its risk level, and Figure 7 shows the results. The *mPEL*-*Q* varied within the range 0.16–0.26, between 0.1 and 1, indicating considerable risk. The *mPEL*-*Q*’s relatively higher value appeared at S5 and S18, which is consistent with the results of the potential ecological risk assessment of *RI*. Thus, it can be determined that the high-value areas of heavy metals in the sampling area are situated at stations S5 and S18.

#### 3.3.4. Risk Assessment Code

In the study by Singh et al. [51], it was revealed that among numerous heavy metal geochemical fractions, changes in acid-soluble fractions caused by human activities influence the bioavailability or mobility of heavy metals in sediment. Thus, the higher the percentage of acid-soluble fraction in geochemical fractions, the higher the migration capacity of heavy metals in sediments, the higher the bioavailability, and the higher the level of potential ecological risk. Figure 7 shows the *RAC* evaluation results of heavy metals in sediments in the Qinjiang River. The mean *RAC* value of each heavy metal decreased in the order of Mn (38.92%) > Zn (13.43%) > Cr (12.68%) > Cu (7.70%) > Fe (1.48%) > Cd (0.75%) > Pb (0.69%) > Co (0.62%) > Ni (0.49%). According to *RAC*, Mn appeared to pose a high risk, Zn and Cr were grouped as medium risk, Fe and Cu were grouped as low risk, and Co, Ni, Cd, and Pb were grouped as no risk. However, it should be noted that the results of Cu are different from those of I_geo_ and *RI*, probably because the *RAC* approach is based on the heavy metals’ geochemical fractions and their proportion to characterize the risk level and does not consider the total concentration of heavy metals, and the bioavailability expressed in the geochemical fractions is unequal to biological toxicity considered by I_geo_ and *RI*. In this study, the total concentration of Cu is higher than the background values; however, Cu is primarily present in the residual fractions, which are more stable and less likely to be released in the sediment and cause harm to the ecological environment.

### 3.4. Source Analysis

Based on the risk assessment of different heavy metals in Qinjiang River sediments, personal correlation analysis, principal component analysis, and cluster analysis were conducted on nine heavy metals in the study area using SPSS25.0 to further investigate the possible sources of heavy metals in Qinjiang River sediments.

#### Principal Component Analysis

**Figure 8:**
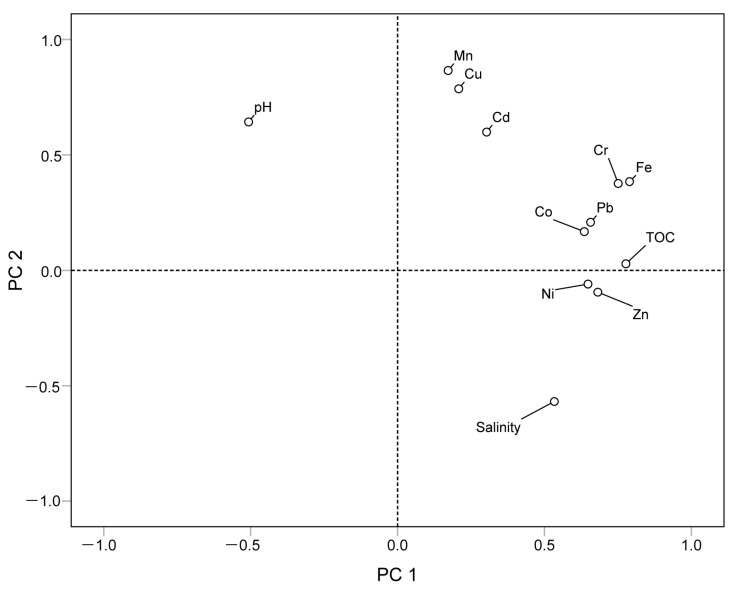
PCA loadings of components 1 and 2 for the nine heavy metals in surface sediments from the Qinjiang River.

Based on personal correlation analysis, the sources of heavy metals were further examined using principal component analysis (PCA) of nine heavy metals. The matrix Kaiser–Meyer–Olkin test value was 0.598, indicating that the data were suitable for PCA (Figure 8). It was observed that the eigenvalues of principal components 1 and 2 are 3.948 and 1.784, respectively, and the variance contribution rates are 43.869 and 19.819, which can better demonstrate the data situation. In the first component (PC1), Fe, Cr, Pb, and Cd demonstrated highly positive loadings, indicating that Fe, Cr, Pb, and Cd may have the same source; in second component (PC2), Cu and Mn have highly positive loadings, suggesting that Cu and Mn may have the same source, and Zn, Co, and Ni have comparable positive loadings, indicating that Zn, Co, and Ni enrichment may be related to Mn.

Table 5 shows the correlations between the nine investigated heavy metals, pH, salinity, and TOC. It was observed that there is a considerable negative correlation between pH and salinity and TOC, suggesting that salinity and TOC decrease with the increase in pH. Fe, Zn, Ni, Cr, Pb, and TOC manifested substantial positive correlations, suggesting that there is a close relationship between the TOC in the soil and total heavy metal concentrations. It was revealed in a study [52] that Fe is less influenced by human interference because of its high concentration, and therefore, it can be employed as a characterization of human and natural factors. In this study, Fe has a considerable positive correlation with Cr and Pb (*p* < 0.01), and therefore, both Cr and Pb are derived from natural factors. Cd has a substantial positive correlation with Fe, Cr, and Pb (*p* < 0.05) and indicates low risk in both the I_geo_ and *RI*, indicating that Cd also belongs to natural factors; Cu and Mn were considerably positively correlated (*p* < 0.01).

**Figure 9:**
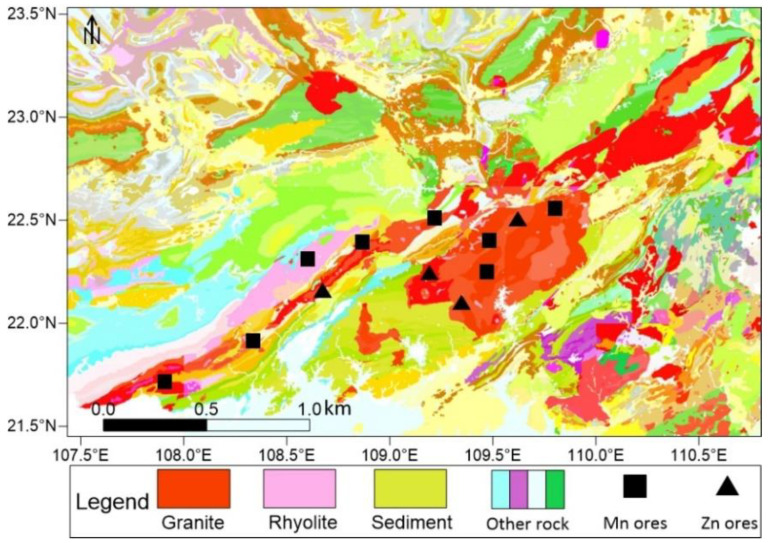
Geological map (1:200,000) and mineral distribution around the Qinjiang River (Adapted with permission from Ref. [56]).

I_geo_ indicated that Mn and Zn were higher than soil background values and had evident characteristics of foreign pollution. PCA and correlation analysis indicated that the two groups of heavy gold denoted by Mn and Zn belong to various sources; the contents of Mn, Cu, and Cd are related to pH, while Zn, Fe, Pb, Co, Ni, and Cr are related to TOC and salinity. Combined with the analysis of heavy metal components, the composition of Mn includes the acid soluble fraction (F1) and the reducible fraction (F2), and the Zn component is the oxidizable fraction (F3). Qinzhou Port is also the largest distribution center of manganese ore in China [53]. The Upper Devonian Liujiang Formation is the primary manganese-bearing strata in the Qinzhou area. Manganese is present in the form of manganese carbonate minerals, such as rhodochrosite, calcite, manganese siderite, and manganese dolomite, while manganese in sediments or weathering crust is in the form of secondary oxides, accompanied by Cu, Cd, and other elements [54]. Zinc deposits in the Qinjiang River Basin occur in the Indo-Hercynian granitic rock mass in Pubei County, Qinzhou City (Figure 9). The zinc deposits are primarily sphalerite, which has a symbiotic relationship with Pb, generating lead–zinc deposits and forming an organic combination with OM and pyrite after weathering and leaching [55].

The survey of soil heavy metals in the Guangxi region showed that the spatial distribution of Cd, As, Cr, Cu, Hg, Ni, Pb, and Zn was mainly controlled by geological background. The content of heavy metals in the soils of carbonate source area is higher than that of clastic source area soils. The enrichment of Cd in surface soils is the result of secondary enrichment and weathering of parent rocks, and the enrichment of other metals is mainly the result of secondary enrichment. During weathering of the carbonate bedrock, the vast majority of the intrinsic heavy metals were leached out, and only 2% of Cd was retained in situ. Soil Fe, Al, Mn oxides, organic carbon, and clay content are closely related to the enrichment of heavy metals [57]. High concentrations of Pb, Cr, Cd, Cu, Zn, As, and Hg in surface sediments were found in Qinzhou Bay, Fangchenggang, and other coastal areas, with heavy metals mainly attributable to industrial sources, including petrochemicals, coal combustion, processing of metals and metal compounds, leather tanning, and human activities: anthropogenic sources accounted for about 70% of all pollution [34]. The sources of heavy metals in atmospheric deposition in the Beibu Gulf region reveal that Pb, Se, and S are mainly derived from coal combustion in coal-fired power plants, Mn, Cd and Hg are largely associated with the possession of Mn mines and the Mn industry, while Zn and Cu in atmospheric deposition are mainly derived from suspended soil particles [58]. Therefore, heavy metal pollution in surface sediments was primarily from manganese ore and zinc ore mining in the Qinjiang River because manganese ore is more common than zinc ore, and the manganese pollution level is higher than that of zinc [59]. Manganese carbonate and its weathered secondary manganese oxide are typically associated with a significant amount of residual Cu and Cd, and their concentrations are usually influenced by pH; that is, high pH is suitable for the deposition and enrichment of these heavy metals, but low pH may lead to acidic dissolved states being formed and migration to the estuarine shelf. Lead–zinc ore and its weathering products generate organic compounds with residual Fe, Co, Cr, and Ni, and their content is related to salinity. A possible reason is that the weathering process of lead–zinc ore causes the conversion of low sulfur into sulfate radicals and the release of associated metal elements.

## 4. Conclusions

The total concentrations of heavy metals were discovered in the decreasing order of Fe > Mn > Zn > Cu > Cr > Pb > Ni > Co > Cd, and they are all significantly lower than the background values, except Mn, Zn, Cr, Cu, and Pb. Cu, Mn, and Zn cause pollution and have certain ecological risks in the Qinjiang River. However, Cu poses less ecological risk than Mn and Zn. Fe, Co, Ni, Cd, Cr, and Pb are not harmful to the environment because of their low content in sediments and natural source heavy metals, but attention must be paid toward their prevention and control. The heavy metal pollution in surface sediments is primarily due to manganese and zinc ore mining in the Qinjiang River. Manganese carbonate and its weathered secondary manganese oxide are typically associated with a significant amount of residual Cu and Cd, and a higher pH is suitable for the deposition and enrichment of these heavy metals. Lead–zinc ore and its weathering products generate organic compounds with residual Fe, Co, Cr, and Ni, and their content is related to salinity.

## Figures and Tables

**Table 1:** Improved BCR sequential extraction procedure.

Step	Extracting Agent	Extraction Process
F1 (Acid Soluble)	20 mL, 0.11 mol/L CH_3_COOH	Shaking at 220 rpm at 22 ± 5 ℃ for 16 h
F2 (Reducible)	20 mL, 0.5 mol/L NH_2_OH-HCl (pH = 1.5)	Shaking at 220 rpm at 22 ± 5 ℃ for 16 h
F3 (Oxidizable)	10 mL, 30% H_2_O_2_; 25 mL, 1.0 mol/L CH_3_COONH_4_ (pH = 2)	Heated 85 ℃ for 1 h. Shaking at 220 rpm at 22 ± 5 ℃ for 16 h
F4 (Residual)	2.5 mL HNO₃, 2.0 mL HF, 1.0 mL HCIO₄	Microwave digestion

**Table 2:** Relationship between potential ecological hazard index and potential ecological hazard level.

$E_{r}^{i}$		Grades of Ecological Risk for a Single Metal	*RI*		Grades of Ecological Risk for a Single Metal
This Study	Zhu et al., 2012 [40]		This Study	Zhu et al., 2012 [40]	
$E_{f}^{i}$ ≤ 30	$E_{f}^{i}$ ≤ 40	Low risk	*RI* ≤ 70	*RI* ≤ 150	Low risk
$30\;<\;E_{f}^{i}$ ≤ 60	$40\;<\;E_{f}^{i}$ ≤ 80	Moderate risk	70 < *RI* ≤ 140	150 < *RI* ≤ 300	Moderate risk
$60\;<\;E_{f}^{i}$ ≤ 120	$80\;<\;E_{f}^{i}$ ≤ 160	Considerable risk	140 < *RI* ≤ 280	300 < *RI* ≤ 600	Considerable risk
$120\;<\;E_{f}^{i}$ ≤ 240	$160\;<\;E_{f}^{i}$ ≤ 320	High risk	*RI* ≥ 280	*RI* ≥ 600	Very high risk
$E_{f}^{i}$ ≥ 240	$E_{f}^{i}$ ≥ 320	Very high risk			

**Table 3:** Values of maximum, minimum, median, average, background (in mg/kg), and coefficient of variation (CV%) for total concentration of heavy metals in the surface sediments from the Qinjiang River.

Elements	Maximum	Minimum	Median	Average	Background	CV
Fe	40,776.19	19,576.20	29,971.10	31,401.95	—	17.66%
Mn	1106.67	147.57	469.66	500.27	159.32	53.55%
Zn	212.44	65.67	101.74	112.49	48.25	37.02%
Co	12.78	6.30	8.63	9.05	14.60	21.08%
Ni	20.79	15.72	17.80	17.82	24.00	9.87%
Cd	0.03	0.01	0.02	0.02	0.07	22.75%
Cr	41.92	13.90	30.49	29.78	21.41	26.49%
Cu	74.52	56.60	65.10	65.45	11.31	7.64%
Pb	33.47	17.08	22.16	22.99	20.43	20.74%

**Table 4:** Percentage of heavy metals in each category associated with biological risks.

Heavy Metals		Fe	Mn	Zn	Co	Ni	Cd	Cr	Cu	Pb
TEL [46,50]		—	460	124	—	15.9	0.68	52.3	18.7	30.2
*PEL* [46,50]		—	1100	271	—	42.8	4.21	160.4	108.2	112.2
The comparison with TEL and *PEL*		% of samples in each guideline								
Ι	<TEL	—	47	19	—	21	100	100	0	85
ΙΙ	>TEL and <*PEL*	—	47	81	—	79	0	0	100	15
ΙΙΙ	>*PEL*	—	6	0	—	0	0	0	0	0

**Table 5:** Pearson correlation matrix for heavy metals concentration in surface sediments from the Qinjiang River.

Elements	Fe	Mn	Zn	Co	Ni	Cd	Cr	Cu	Pb	pH	Salinity	TOC
Fe	1.000											
Mn	0.405 *	1.000										
Zn	0.279	0.012	1.000									
Co	0.481 *	0.157	0.601 **	1.000								
Ni	0.254	0.202	0.491 *	0.459 *	1.000							
Cd	0.390 *	0.479 *	0.234	0.331	0.045	1.000						
Cr	0.879 **	0.405 *	0.389 *	0.353	0.280	0.393 *	1.000					
Cu	0.519 *	0.729 **	0.007	0.053	0.141	0.290	0.576 **	1.000				
Pb	0.631 **	0.158	0.339	0.532 **	0.342	0.457*	0.425 *	0.106	1.000			
pH	−0.350	0.433 *	−0.170	−0.055	−0.126	0.200	−0.251	0.397 *	−0.200	1.000		
Salinity	0.169	-0.341	0.369	−0.005	0.461 *	−0.155	0.305	−0.117	0.056	−0.581 **	1.000	
TOC	0.620 **	0.294	0.446 *	0.370	0.511 *	0.116	0.526 *	0.219	0.402 *	−0.394 *	0.334	1.000

Note: * Correlation is significant at the 0.05 level. ** Correlation is significant at the 0.01 level.

## Data Availability

The data are contained within the article.
